# Vitamin D_3_-dependent VDR signaling delays ron-mediated breast tumorigenesis through suppression of β-catenin activity

**DOI:** 10.18632/oncotarget.4059

**Published:** 2015-05-10

**Authors:** Abby L. Johnson, Glendon M. Zinser, Susan E. Waltz

**Affiliations:** ^1^ Department of Environmental Health, University of Cincinnati, Cincinnati, Ohio, USA; ^2^ Department of Cancer Biology, University of Cincinnati College of Medicine, Cincinnati, Ohio, USA; ^3^ Department of Research Service, Cincinnati Veterans Affairs Medical Center, Cincinnati, Ohio, USA

**Keywords:** vitamin D_3_ receptor, ron, β-catenin, breast cancer

## Abstract

The Ron receptor is upregulated in human breast cancers and correlates with enhanced metastasis and reduced patient survival. Ron overexpression drives mammary tumorigenesis through direct β-catenin activation and augmented tumor cell proliferation, migration and invasion. Ron and β-catenin are also coordinately elevated in breast cancers. The vitamin D receptor (VDR) antagonizes β-catenin signaling. Herein, we examined mammary tumor onset and progression using a Ron-driven murine model of breast tumorigenesis crossed with VDR deficient mice. VDR ablation accelerated mammary tumor onset and led to tumors that exhibited a desmoplastic phenotype and enhanced metastases. Tumor levels of active β-catenin were markedly increased in the absence of VDR. *In vitro*, VDR activation in breast cancer cells reduced β-catenin activation and transcriptional activity leading to elevated expression of the extracellular Wnt inhibitor dickkopf-related protein 1, and a reduction in the interaction of β-catenin with the cyclin D1 promoter. Expression of a stabilized form or β-catenin ablated the protective effects of VDR activation. Collectively, these studies delineate a protective role for VDR signaling in Ron-induced mammary tumorigenesis through disruption of β-catenin activation.

## INTRODUCTION

The Ron receptor tyrosine kinase, also known as macrophage-stimulating 1 receptor (MST1R), is overexpressed in approximately 50% of human breast cancers [1] and is associated with increased metastasis and poor patient prognosis [2]. Similarly, overexpression of hepatocyte growth factor-like protein (HGFL), the only known Ron ligand, and matriptase, an enzyme involved in the cleavage of pro-HGFL to produce the active ligand, are also negative prognostic factors in breast cancer [3]. Ron expression is primarily localized to epithelial cells and resident macrophages (reviewed in [4]). In normal mammary epithelial cells, Ron expression is very low but becomes overexpressed in breast cancer cells [1]. In Ron expressing human breast cancer cell lines, T47D and ZR-75-1, activation of the Ron receptor increases cell proliferation, migration and invasion [1, 5].

Oncogenic Ron activation is associated with the activation of a variety of signal transduction pathways including activation of PI3-K/Akt [6, 7], MAPK [8], Ras [9, 10], Src, and β-catenin [11, 12]. In a model of breast cancer with targeted overexpression of Ron in the mammary epithelium, β-catenin was shown to be required for Ron-mediated mammary tumorigenesis [13, 14]. The β-catenin signaling axis has been shown to be upregulated in many human breast cancers and is associated with poor disease prognosis [15-20]. Activation of β-catenin can occur directly through Ron-induced phosphorylation of residues Tyr 654 and Try 670 resulting in β-catenin nuclear translocation and enhanced transcription of cyclin D1 and c-Myc target genes [14]. Moreover, genetic ablation of β-catenin reduced Ron-directed mammary tumor growth and metastasis, as well as cell proliferation *in vitro* [14], which was further confirmed in a study utilizing a transgenic mouse model with Ron overexpression in the mammary epithelium and targeted deletion of β-catenin [21]. Thus, understanding how perturbations in the Ron-β-catenin signaling axis affect the therapeutic response of breast cancers with a highly metastatic phenotype is critical for lessening the deaths associated with metastatic disease.

The vitamin D receptor (VDR) is a nuclear hormone receptor that protects against mammary tumor formation, progression and metastasis in the presence of 1,25D_3_ through induction of cell-cycle arrest, promotion of apoptosis, regulation of differentiation, and reduction in angiogenesis (reviewed in [22-25]). Ligand-dependent VDR signaling has been shown to antagonize the β-catenin signaling pathway through several mechanisms in human and murine colon cancer cells (reviewed in [26]). For example, induced VDR activation promotes transcriptional upregulation of E-cadherin which inhibits β-catenin nuclear localization and induces translocation to adherins junctions at the plasma membrane [27-29]. Ligand-dependent VDR signaling also increases mRNA expression of Dickkopf-related protein 1 (DKK-1), an inhibitor of the canonical Wnt signaling pathway, that subsequently prevents the activation of β-catenin by way of Wnt signaling [30]. TCF-4 is transcriptionally regulated by VDR in murine colon and breast cancer cells for growth inhibition [31]. In colon cancer, ligand-bound VDR also competes with transcription factors for β-catenin binding between the activator function-2 (AF-2) domain of VDR and the C terminus of β-catenin [32] thus physically preventing transcriptional activation of TCF/LEF target genes [27]. Given the negative regulation of β-catenin signaling by VDR activation, we hypothesized that vitamin D_3_-dependent VDR signaling would impede the aggressive progression of Ron-mediated breast tumorigenesis.

Herein we demonstrate that VDR limits Ron-induced mammary tumor initiation and growth by decreasing active β-catenin levels and through a reduction in β-catenin target genes. Further, vitamin D_3_ treatment reduced breast cancer cell growth, migration, and invasion in Ron expressing breast cancer cells. Moreover, Ron knockdown (KD) further sensitized breast cancer cells to the growth inhibitory effects of vitamin D_3_, while constitutive activation of β-catenin reverted the effects of vitamin D_3_. Mechanistically, 1,25D_3_ reduced active β-catenin levels, decreased β-catenin transcriptional activity, increased expression of DKK-1, and diminished the association of active β-catenin with the cyclin D1 promoter. Thus, combinational therapies integrating Ron or receptor tyrosine kinase (RTK) antagonists with vitamin D_3_ or potent vitamin D_3_ analogs in breast cancers exhibiting Ron overexpression may provide a beneficial alternative to current standard of care chemotherapeutic agents.

## RESULTS

### Loss of VDR signaling accelerates epithelial hyperplasia in the mammary glands of MMTV-Ron mice

**Figure 1:**
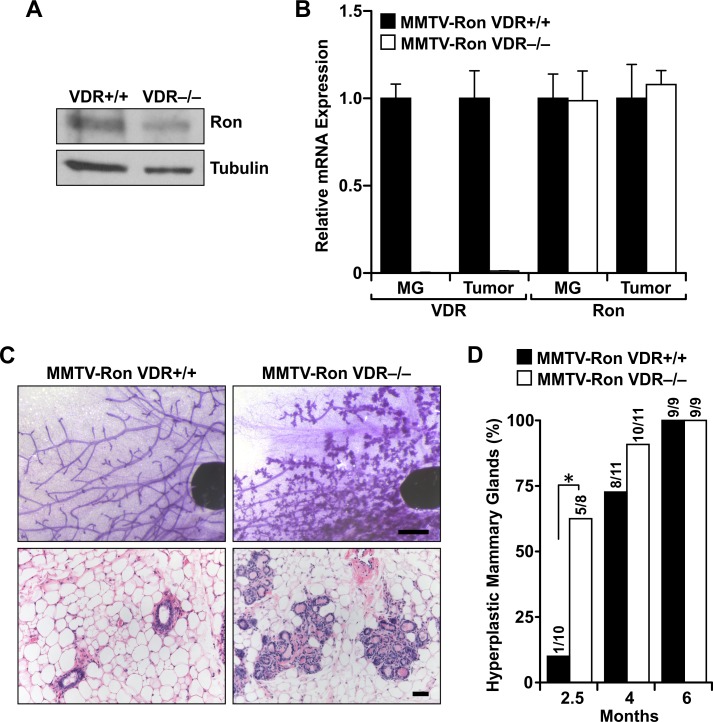
VDR signaling delays Ron-mediated mammary gland hyperplasia **A.** Western blot of mammary lysates from MMTV-Ron VDR+/+ and VDR−/− mice depicting Ron expression levels. **B.** qRT-PCR analysis of Ron and VDR mRNA expression in mammary glands (MG) and tumors from MMTV-Ron VDR+/+ and VDR−/− mice. Data represent mean values from three independent experiments ± SE. **C.** Representative mammary whole mounts (upper panels) and H&E-stained tissue sections (lower panels) from 10 week-old MMTV-Ron VDR+/+ and VDR−/− mammary glands (*n* = 8-11). **D.** Incidence of mice with hyperplastic mammary glands at 2.5, 4 and 6 months of age from MMTV-Ron VDR+/+ and VDR−/− animals. *P < 0.05.

To examine the role of vitamin D receptor (VDR) signaling in the development of Ron-mediated breast tumorigenesis, MMTV-Ron transgenic mice were crossed to mice deficient for VDR (VDR−/−) generating offspring with VDR haploinsufficiency. Littermates were intercrossed to generate VDR+/+, VDR−/− mice or mice heterozygous for a functional VDR with Ron overexpression specific to the mammary epithelium. Analysis of protein and mRNA from transgenic mouse mammary gland lysates shows Ron and VDR expression levels in the MMTV-Ron VDR−/− model (Figures 1A and 1B).

Previous studies demonstrated that tissues with targeted overexpression of Ron in the mammary epithelium develop hyperplasia by 12 weeks of age [13]. Mammary gland hyperplasia was evident in the majority of MMTV-Ron VDR−/− mice at 10 weeks (Figures 1C and 1D). Contrastingly, most age-matched glands from MMTV-Ron VDR+/+ mice exhibited a normal phenotype at this early time point suggesting that VDR delays Ron-mediated mammary hyperplasia. However, by 4 months of age almost all MMTV-Ron VDR+/+ and VDR−/− mice exhibited severely dilated, cystic acini similar to the mammary epithelium previously reported in MMTV-Ron induced tumorigenesis [13].

### VDR signaling delays mammary tumor onset and reduces metastasis to the lungs and liver

**Figure 2:**
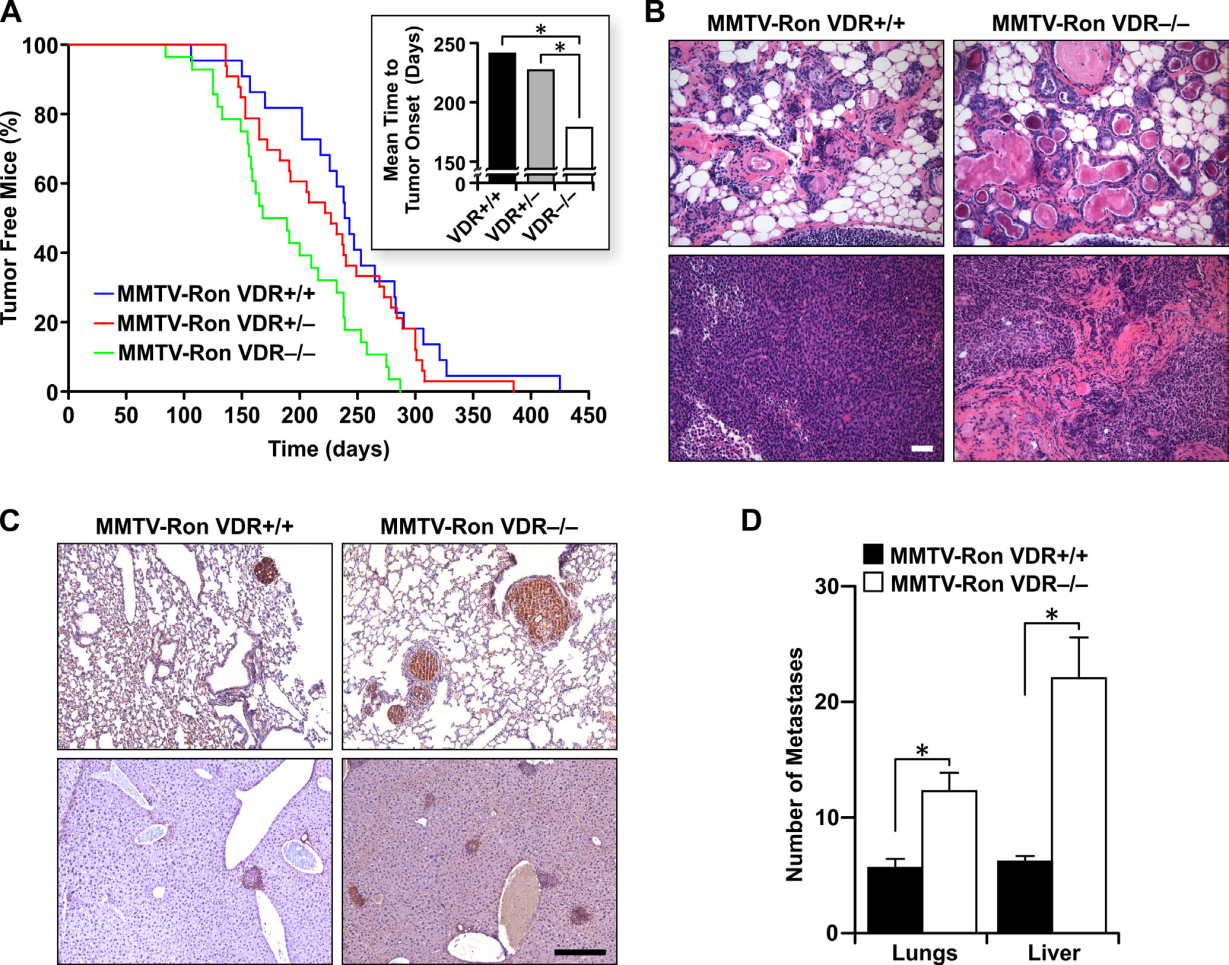
VDR signaling delays mammary tumor formation and alters disease progression in MMTV-Ron mice **A.** Mammary tumor kinetics from MMTV-Ron VDR+/+, VDR+/−, and VDR−/− mice. Between 22 and 33 female mice per genotype were examined temporally for palpable tumor formation. The percent tumor-free mice is plotted as a function of age and is statistically significant with complete VDR ablation compared to VDR haploinsufficiency and VDR wild type mice. Inset: The mean time-to-tumor onset was plotted for each genotype. **B.** Representative mammary glands (upper panels) and tumors (lower panels) from 8 month-old MMTV-Ron VDR+/+ and VDR−/− mice showing enhanced ductal dilation and desmoplasia with VDR ablation (*n* = 9-11 per genotype). **C.** Representative immunohistochemical staining for Ron in lung (upper panels) and liver (lower panels) sections from 10 month-old MMTV-Ron VDR+/+ and VDR−/− mice showing more metastases with loss of VDR (n = 5-6 per genotype). **D.** The average number of metastases per lung (*n* = 24-25) and liver (*n* = 14-19) per genotype as assessed by histological analysis is plotted. Data represent mean values ± SE. *P < 0.05.

Mammary tumor formation occurred in all study mice regardless of VDR status. However, MMTV-Ron mice homozygous or heterozygous for VDR exhibited significantly longer times to palpable tumor formation compared to VDR−/− mice (Figure 2A and inset). The mean time-to-tumor onset was 178.5 days for VDR−/− mice compared to 227 days for VDR+/− and 241 days for VDR+/+ mice in the MMTV-Ron background. Morphological assessment of mammary tissue from eight month-old transgenic mice displayed extremely dilated ducts in the absence of VDR with large foci of keratin pearls containing concentric circles of abnormal squamous cells with a high deposition of cytokeratins (Figure 2B, upper panels) and MMTV-Ron VDR−/− tumors demonstrated a desmoplastic phenotype, densely abundant in connective tissue (Figure 2B, lower panels).

VDR signaling has been shown to inhibit the metastatic potential of human and mouse breast cancer cells [33]. Tumor progression was similarly monitored in MMTV-Ron VDR+/+ and MMTV-Ron VDR−/− mice with nearly 90% of all study animals exhibiting lung and liver metastases. While VDR expression only modestly delayed the onset of lung and liver metastases (data not shown), the number of metastases per mouse tissue was consistently greater in MMTV-Ron VDR−/− mice compared to controls as seen by the representative immunohistochemical staining for Ron in the lungs (Figure 2C upper panels) and liver (Figure 2C lower panels). When quantified, the number of metastases per tissue was significantly increased with VDR ablation (Figure 2D).

### Ron-induced β-catenin signaling is reduced in the presence of a functional VDR

**Figure 3:**
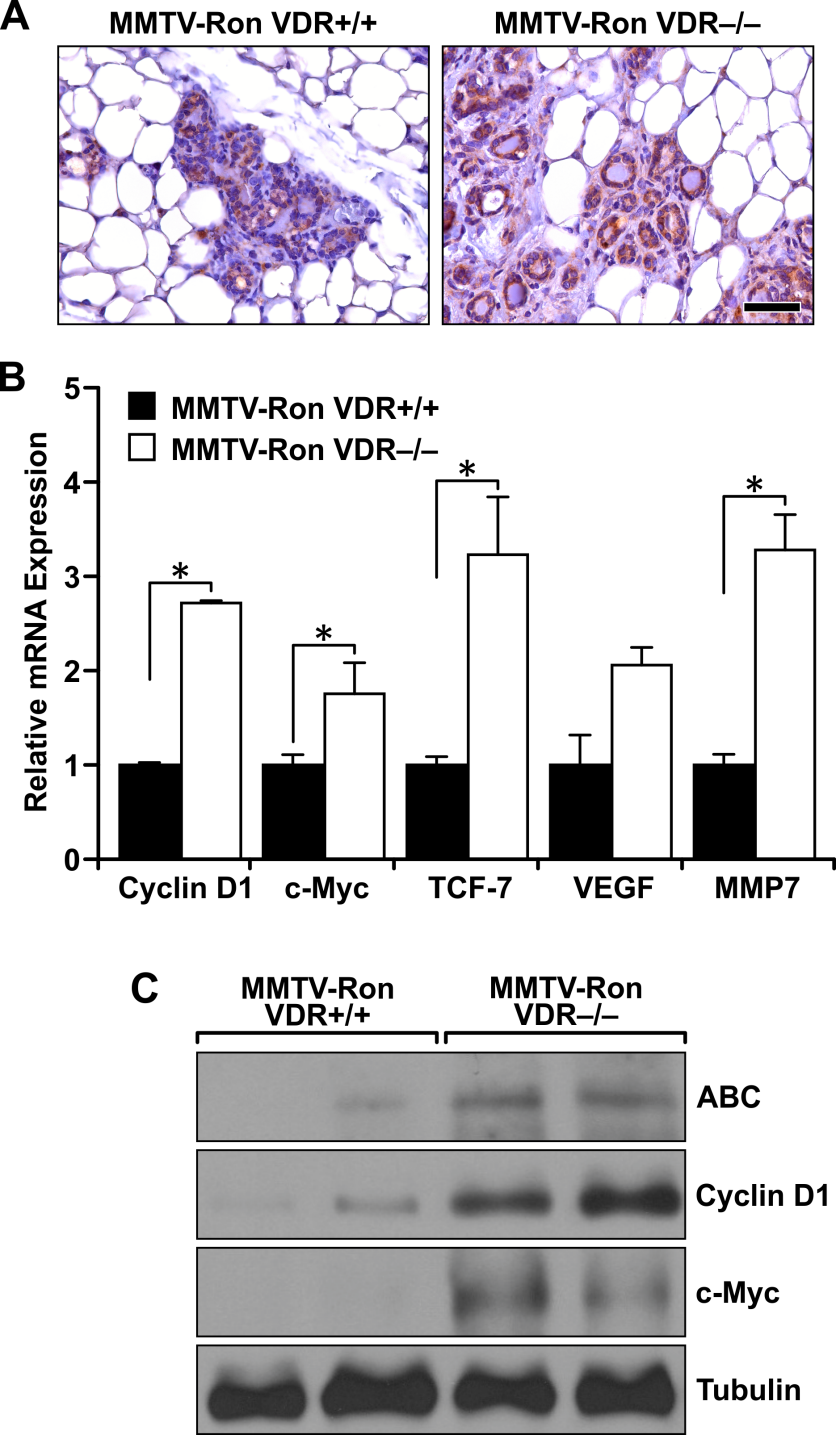
Enhanced downstream β-catenin signaling in Ron-mediated mammary tumorigenesis with VDR ablation **A.** Representative immunohistochemical staining for β-catenin in MMTV-Ron VDR+/+ and VDR−/− mammary glands from 4 month-old mice demonstrating enhanced expression in the absence of VDR (*n* = 6 per genotype). **B.** qRT-PCR mRNA expression of β-catenin target genes Cyclin D1, c-Myc, TCF-7, VEGF, and MMP7 in mammary tumors from 8 month-old MMTV-Ron VDR+/+ and VDR−/− mice. Data represent mean values from three independent experiments ± SE. **C.** Western analysis demonstrating enhanced expression of active β-catenin (ABC) levels in two representative MMTV-Ron VDR−/− tumor lysates compared to two MMTV-Ron VDR+/+ tumors that correlates with increased expression of β-catenin target genes cyclin D1 and c-Myc. *P < 0.05.

β-catenin is a well-established downstream target of Ron signaling, and upregulation of both pathways correlates with aggressive breast tumorigenesis and a high metastatic potential. Therefore, we analyzed the local expression of β-catenin within mammary tumors of the MMTV-Ron VDR+/+ and VDR−/− mice (Figure 3A). Expression of β-catenin within the VDR−/− mammary epithelium was abundant and ubiquitous as demonstrated by both dark cytoplasmic and nuclear staining compared to tissues with a functional VDR that had more selective and localized β-catenin expression in the cytoplasm. qRT-PCR analysis of mammary tumors demonstrated increased β-catenin transcriptional activity as judged by enhanced mRNA expression of β-catenin target genes (Cyclin D1, c-Myc, TCF-7, VEGF and MMP7) in the absence of VDR (Figure 3B). This was further verified by Western analysis in the MMTV-Ron VDR−/− mammary tumors showing enhanced active β-catenin (ABC) expression, correlating with higher protein levels of c-Myc and Cyclin D1 (Figure 3C).

### Ligand-dependent VDR-mediated inhibition of growth, migration and invasion is modulated by Ron receptor status

**Figure 4:**
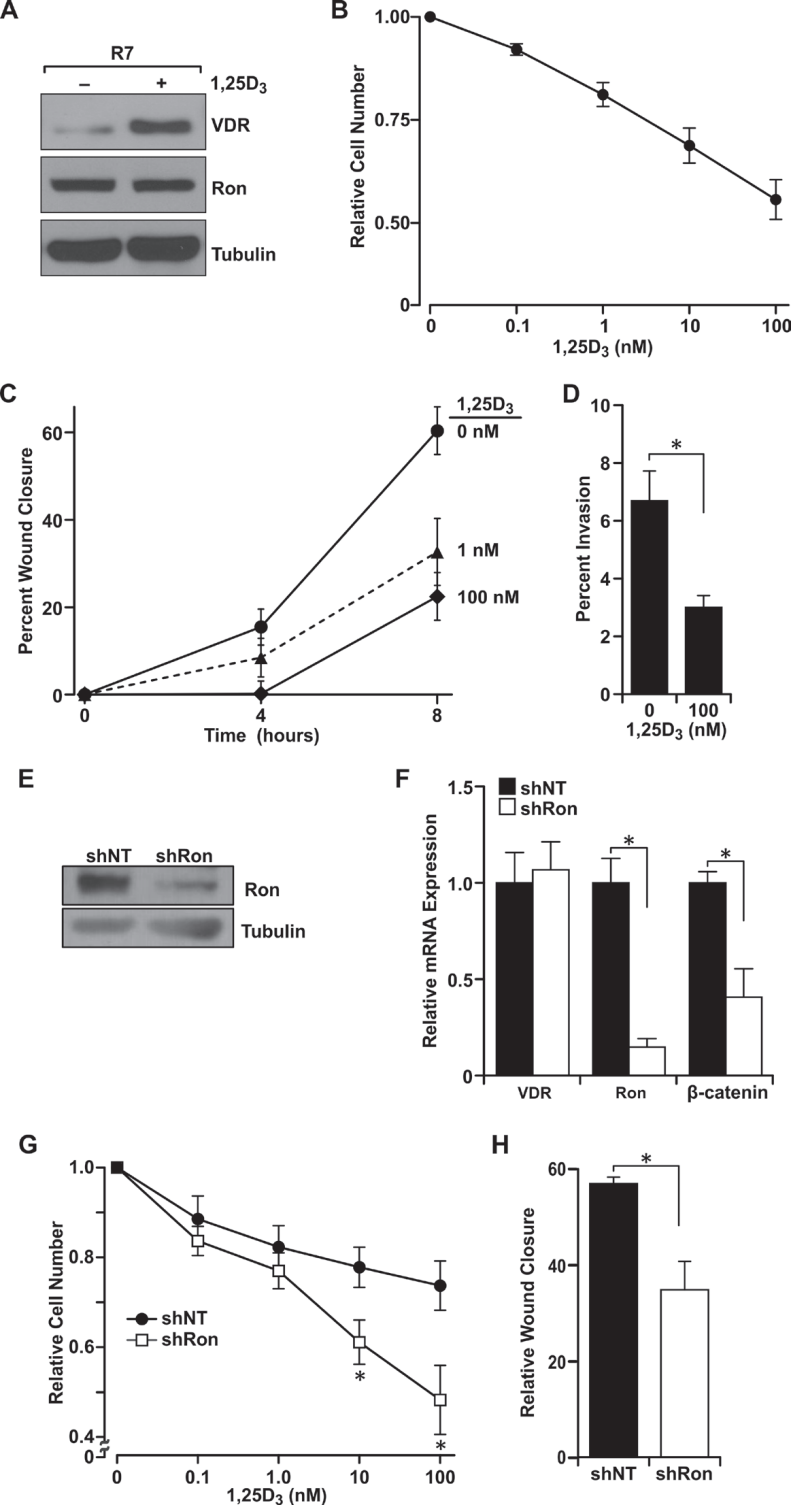
Ron receptor status determines epithelial cell sensitivity to vitamin D_**3**_-mediated growth inhibition and delays in migration and invasion **A.** Western analysis of levels of VDR and Ron ± 100 nM 1,25D_3_ in R7 cells. **B.** R7 cells were treated with the designated concentrations of 1,25D_3_ for 72 hours and cell viability/number was determined by crystal violet assays. Data are normalized to vehicle treated cells set at 1 and represent mean values from three independent experiments ± SE. **C.** R7 cells treated with the designated concentrations of 1,25D_3_ starting at the 0 hour time point were examined in scratch assays for the percent of gap closure after 4 and 8 hours. Data represent mean values from three independent experiments ± SE. **D.** Matrigel invasion assay with R7 cells in the presence or absence of 100 nM 1,25D_3_. Data represent mean values from four independent experiments ± SE. **E.** Western blot demonstrating the reduction in Ron expression in T47D cells stably transfected with a shRon lentivirus. **F.** qRT-PCR analysis of VDR, Ron and β-catenin mRNA expression in control T47D cells (shNT) and Ron knockdown T47D cells (shRon). Data represent mean values from three independent experiments ± SE. **G.** MTT assays of T47D control (shNT) and shRon cells treated with the designated concentrations of 1,25D_3_ for 48 hours. Data represent mean values from 3-4 independent experiments per cell line ± SE. **H.** Scratch assays in T47D control (shNT) and shRon cells treated with 100 nM 1,25D_3_ for 24 hours. Data represent mean values normalized to the vehicle-treated control group of each cell type and are from three independent experiments ± SE. *P < 0.05.

Murine tumor cells derived from MMTV-Ron mice (R7) mimic the Ron overexpression observed in human breast cancers and serve as an excellent model for studying Ron signaling in breast tumorigenesis [13]. In Figure 4A, we demonstrate that these cells express VDR and exhibit characteristic increases in VDR expression in response to 1,25D_3_-stimluation. To examine VDR functionality, we show that vitamin D_3_ treatment was able to reduce the rapid growth of R7 cells in a concentration-dependent manner, with nearly 50% reduction observed at 100 nM 1,25D_3_ (Figure 4B).

As mammary tumors from MMTV-Ron mice undergo rapid growth and metastasis [13], we next measured the migratory capability of R7 cells in the presence of increasing concentrations of 1,25D_3_. Vitamin D_3_ in its active form dose-dependently delayed wound closure indicative of slower migration after 8 hours (Figure 4C). We next measured R7 cell invasiveness in the presence or absence of 1,25D_3_. At 100 nM 1,25D_3_, the percent of breast cancer cell invasion was significantly reduced (Figure 4D).

To examine these effects in human breast cancer cells, T47D cells, with and without a Ron knockdown (KD) were utilized [14]. Ron KD was confirmed by Western and qRT-PCR analyses (Figures 4E and 4F). Ron KD (shRon) in T47D cells correlated with decreased β-catenin mRNA expression (Figure 4F) and active β-catenin protein levels (data not shown), while VDR mRNA transcript levels were comparable between the non-transfected control (shNT) and shRon T47D cells. 1,25D_3_ dose-dependently reduced T47D cell numbers with roughly a 25% reduction at a concentration of 100 nM (Figure 4G). However, diminution of Ron expression sensitized T47D cells to vitamin D_3_-induced growth inhibition as shRon T47D cell number was further and significantly reduced an additional 16.7% and 25.4% at 10 nM and 100 nM 1,25D_3_, respectively compared to shNT controls (Figure 4G). To account for the effects of Ron loss on cell growth, cell number was normalized to 1 for each cell type independently in Figure 4G.

Similar to R7 cells, 1,25D_3_ also delayed wound closure in T47D cells indicative of a reduced migratory ability in the presence of vitamin D_3_. Compared to shNT cells, Ron KD significantly delayed the rate of cell migration suggesting the importance of Ron signaling in driving the enhanced migratory phenotype (data not shown). However, compared to the respective vehicle-treated control, 1,25D_3_ significantly delayed wound closure further in shRon T47D cells after 24 hours compared to shNT controls, suggesting that the effect of vitamin D_3_ on tumor cell migration is influenced by Ron receptor status (Figure 4H).

### Vitamin D_3_ decreases β-catenin transcriptional activity and growth promotion

**Figure 5:**
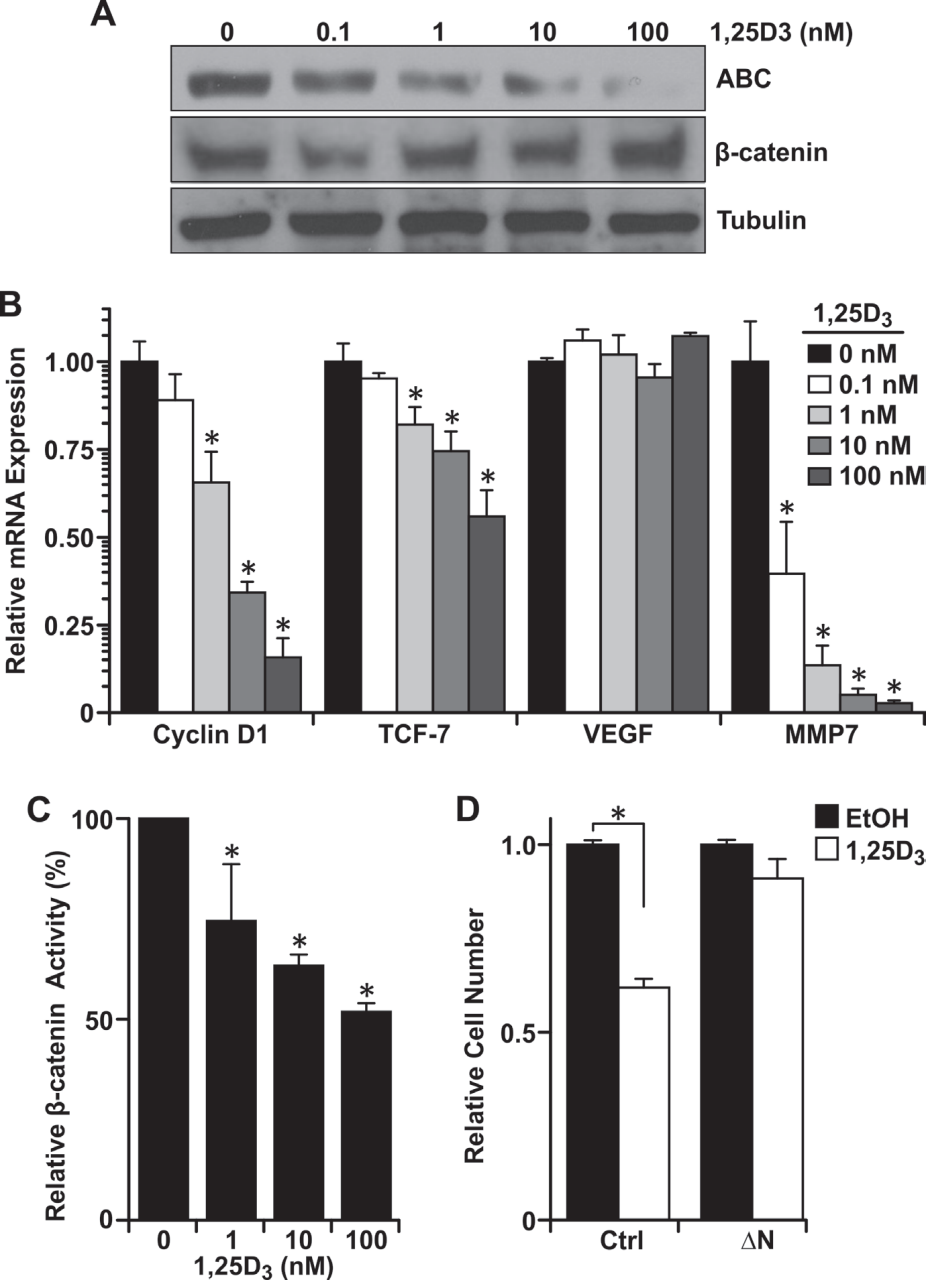
Vitamin D_**3**_-dependent VDR signaling reduces β-catenin transcriptional activity **A.** Western analysis demonstrating a dose-dependent reduction in active β-catenin (ABC) levels in R7 cells treated with the designated concentrations of 1,25D_3_ for 48 hours, but no change in total β-catenin. **B.** qRT-PCR analysis of β-catenin target genes cyclin D1, TCF-7, VEGF, and MMP7 in R7 cells treated with the designated concentrations of 1,25D_3_ for 72 hours. Data represent mean values from three independent experiments ± SE. **C.** Dual luciferase assays in R7 cells co-transfected with Topflash and pRLTX (expressing *Renilla*) plasmids for 48 hours, then treated with the designated concentrations of 1,25D_3_ for 24 hours. Luciferase activities were normalized for transfection efficiency to *Renilla* activity. Data represent mean values from three independent experiments ± SE. **D.** Crystal violet staining of R7 cells transfected with a stabilized form of β-catenin (ΔN) or empty vector and treated with 100 nM 1,25D_3_ or vehicle control (EtOH). Data represent mean values from four independent experiments ± SE. *P < 0.05.

To understand the mechanism of vitamin D_3_-dependent reductions in growth, migration and invasion in cells with Ron overexpression, we first measured protein levels of active β-catenin in R7 cells treated with various concentrations of 1,25D_3_ (Figure 5A). Vitamin D_3_ reduced active β-catenin levels without affecting total levels of β-catenin. As protein levels of active β-catenin decreased in response to 1,25D_3_, decreased mRNA levels of the well-established β-catenin target genes cyclin D1, TCF-7, and MMP-7 were also observed (Figure 5B). The inability of vitamin D_3_ or VDR to significantly regulate VEGF expression in R7 cells or MMTV-Ron tumors, respectively, suggests that VDR signaling may not play a role in mitigating angiogenesis in this tumor model, but future work is warranted to satisfy this claim. To verify that β-catenin transcriptional activity was reduced with increasing concentrations of 1,25D_3_, we performed a dual luciferase assay in R7 cells utilizing reporter plasmids containing multiple copies of the TCF binding sequence, then treated the cells with increasing concentrations of 1,25D_3_ for 24 hours post transfection. Transcriptional activity of β-catenin was negatively modulated by vitamin D_3_ over time as increasing concentrations decreased luciferase activity (Figure 5C).

To test whether 1,25D_3_-dependent VDR signaling inhibits β-catenin-induced growth promotion, we transfected R7 cells with a plasmid encoding a stabilized form of β-catenin (ΔN) [34]. Interestingly, the ability of 1,25D_3_ to inhibit growth in non-transfected R7 controls (NT) was almost completely rescued in R7 cells with the ΔN construct (Figure 5D). To account for the growth promoting effects of the ΔN construct, cell number was normalized to the respective vehicle-treated control in Figure 5D. This suggests that 1,25D_3_ inhibits R7 cell growth through suppression of active β-catenin levels and downstream transcriptional activity.

### VDR activation inhibits β-catenin signaling by increasing DKK-1 expression and by displacing the transcriptional co-activator from binding sequences within the cyclin D1 promoter

**Figure 6:**
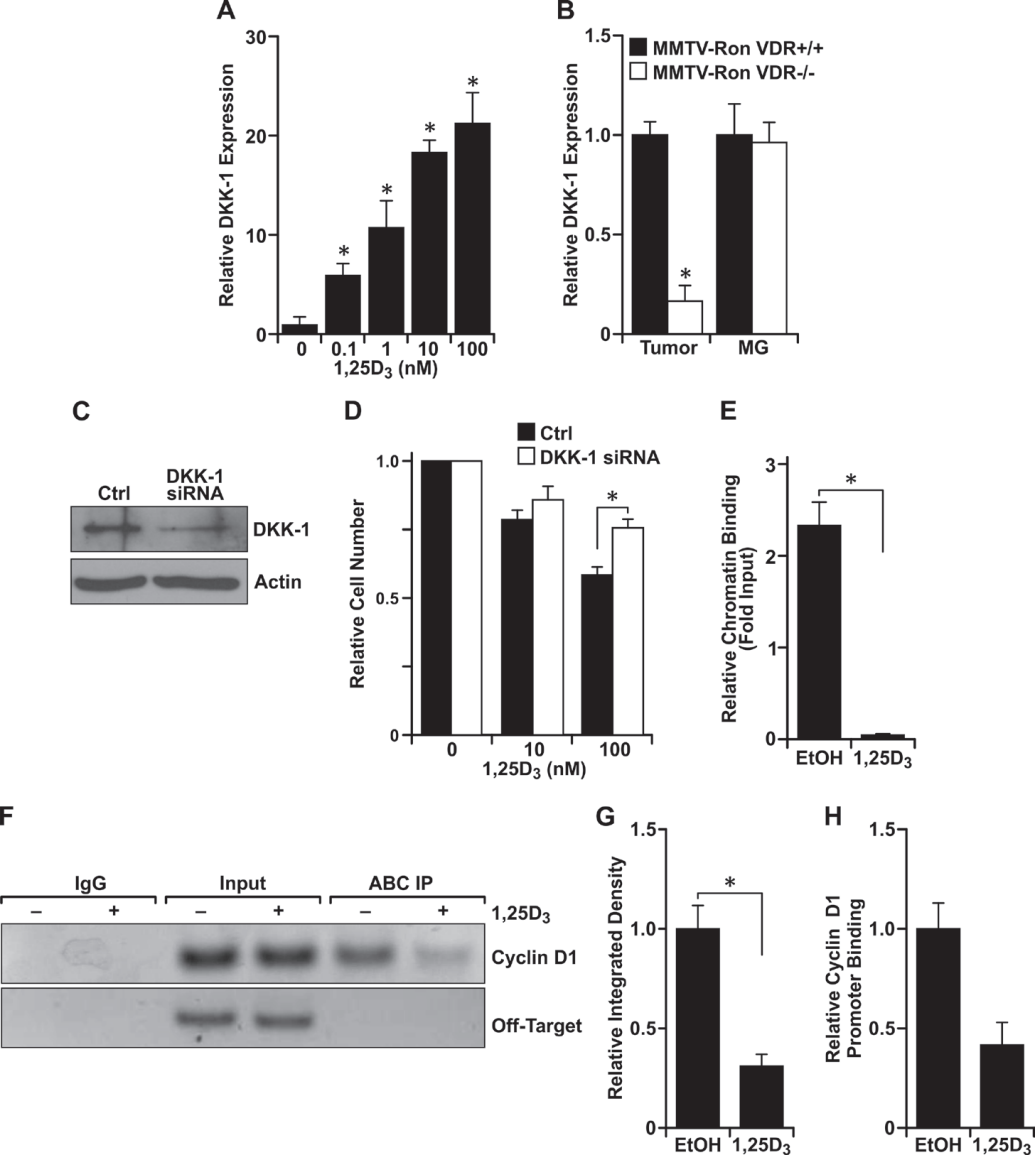
Vitamin D_**3**_-dependent VDR signaling induces DKK-1 expression and binds to β-catenin to disrupt interaction at consensus sequences within promoters of TCF/LEF target genes **A.** qRT-PCR mRNA expression of DKK-1 in R7 cells treated with the designated concentrations of 1,25D_3_ for 72 hours. Data represent mean values from three independent experiments ± SE. **B.** qRT-PCR mRNA expression of DKK-1 in tumors and mammary glands (MG) from 8 month-old MMTV-Ron VDR+/+ and VDR−/− mice demonstrating a reduction in DKK-1 levels in tumors with loss of VDR. Data represent mean values from three independent experiments ± SE. **C.** Western blot demonstrating loss of DKK-1 protein expression with siRNA-mediated silencing in R7 cells. **D.** R7 cells transfected with siRNA against DKK-1 were treated with the designated concentrations of 1,25D_3_ for 72 hours and cell viability/number was determined by crystal violet assays. Data are normalized to the respective vehicle treated cells set at 1 and represent mean values from two independent experiments performed in quadruplicate ± SE. **E.** Chromatin immunoprecipitation (ChIP) assays with a mouse IgG isotype control and an anti-active β-catenin (ABC) antibody. ChIP-ABC quantitative real time PCR (qRT-PCR) analysis of R7 cells treated with 100 nM 1,25D_3_ for 72 hours. The graph shows qRT-PCR on DNA purified from ChIP-ABC, using primers designed to the LEF-1 binding sequence within the mouse cyclin D1 promoter and relative to the respective input controls. Vitamin D_3_ treatment significantly reduces enrichment compared to the vehicle control (EtOH). Data represent mean values from three independent experiments ± SE. **F.** Representative agarose gel from ChIP-ABC PCR showing reduced cyclin D1 promoter enrichment with vitamin D_3_ treatment in R7 cells. Negative control primers designed to a site 4000 bp upstream of the LEF-1 binding sequence within the cyclin D1 promoter (Off-Target) verify specificity of cyclin D1 primers to the sheared DNA product. **G.** Densitometry analysis of PCR products from three separate ChIP-ABC experiments supporting loss of ABC interaction with the cyclin D1 promoter. Error bars represent SE. **H.** Re-ChIP qRT-PCR analysis of R7 cells treated with 100 nM 1,25D_3_ for 72 hours and sequentially immunoprecipitated with anti-ABC then anti-VDR antibodies. The graph shows qRT-PCR of DNA purified from ChIP-ABC-VDR, using primers designed to the LEF-1 binding sequence within the mouse cyclin D1 promoter, and showing less interaction of the ABC-VDR complex at the cyclin D1 promoter. Data represent the relative mean CT values from two experiments ± standard deviation. *P < 0.05.

Although treatment with 1,25D_3_ for 24 hours significantly reduced β-catenin transcriptional activity (Figure 5C), treatment for 0.5, 1 or 6 hours had no significant effect on luciferase reporter activity (data not shown), suggesting that 1,25D_3_ and VDR regulate β-catenin transcriptional activity through a VDR-mediated transcriptional component. As a well-established transcriptional target of VDR and an extracellular inhibitor of canonical Wnt signaling upstream of β-catenin, Dickkopf-related protein 1 (DKK-1) mRNA levels were upregulated with increasing concentrations of 1,25D_3_ in R7 cells (Figure 6A). DKK-1 mRNA levels from MMTV-Ron tumors but not mammary glands were also reduced with VDR ablation (Figure 6B). To verify the functional significance of elevated DKK-1 in 1,25D_3_-treated cells, cell growth in R7 cells with siRNA directed silencing of DKK-1 was measured (Figure 6C). Targeted knockdown of DKK-1 significantly rescued the 1,25D_3_-mediated growth inhibition (Figure 6D). Collectively, our data suggest that DKK-1 is a transcriptional target of VDR that plays a role in regulating cell growth through the inhibition of canonical Wnt/β-catenin signaling. Of note, however, as DKK1 mRNA levels were found to be upregulated after 48 hours following 1,25D_3_-treatment, DKK1 is likely not involved in mediating migratory defects observed in R7 cells after 8 hours of 1,25D_3_ exposure.

Immunoprecipitation studies were performed and demonstrated an interaction between active β-catenin and VDR in R7 cells (data not shown). Therefore, we hypothesized that the ligand-dependent VDR/active β-catenin interaction prevents β-catenin from binding to TCF/LEF consensus sequences with the promoters of its target genes. Therefore, a chromatin immunoprecipitation assay was performed utilizing an anti-active β-catenin antibody and primers designed to the LEF-1 binding sequence within the mouse cyclin D1 promoter. Treatment of R7 cells with 100 nM 1,25D_3_ significantly reduced enrichment of immunoprecipitated DNA as measured by qRT-PCR and densitometry of PCR results from agarose gels (Figures 6E, 6F and 6G). Thus, vitamin D_3_ disrupts the interaction of active β-catenin with the cyclin D1 promoter in breast cancer cells. To assess the effect of vitamin D_3_ on VDR-bound β-catenin at the cyclin D1 promoter, we next conducted sequential ChIP assays (Re-ChIP) using an antibody to pull-down active β-catenin, eluted the antibody/agarose complex from protein and chromatin, and then performed a second immunoprecipitation with an anti-VDR antibody. Similarly, we found that treatment with 1,25D_3_ effectively reduced the interaction of the VDR/active β-catenin complex with the LEF-1 sequence within the cyclin D1 promoter (Figure 6H).

## DISCUSSION

Ron expression is abnormally high in about 50% of primary human breast carcinomas [1]. Previous studies have shown that Ron-mediated breast tumorigenesis requires β-catenin activation and transcriptional activity for tumor formation, cell proliferation and metastasis [13, 14]. Due to the antagonistic role of VDR on β-catenin signaling in human colon cancers, in this study the role of VDR in Ron-induced mammary tumorigenesis was investigated. Our studies provide the first *in vivo* evidence for the protective role of VDR in Ron-induced tumor initiation and progression in the breast by mitigating the transcriptional activity of β-catenin.

While all MMTV-Ron expressing study animals eventually developed hyperplasia with ductal ectasia and regression of tertiary branches, complete loss of VDR signaling was associated with earlier mammary acinar hyperplasia, seen in mice as young as 10 weeks of age. Similarly, MMTV-Ron VDR−/− mice exhibited significantly shorter tumor latency than VDR heterozygous and homozygous littermates. Together, our *in vivo* data suggest that the presence of at least one VDR allele participates to delay Ron-induced mammary tumorigenesis.

To understand the role of VDR in the regulation of Ron-induced tumorigenesis, we utilized R7 mammary tumor cells and the human breast cancer cell line, T47D, with and without a targeted Ron knockdown (KD). 1,25D_3_ has previously been shown to inhibit the growth of a variety of normal and transformed epithelial cells, including T47D cells (reviewed in [35-37]). In concurrence, we demonstrate the potent regulatory effects of 1,25D_3_ on R7 and T47D cell number, migration and invasion despite the high Ron expression in both cell lines [14]. Additionally, Ron KD significantly reduced T47D cell number and migration even more than shNT controls at high concentrations of vitamin D_3_ suggesting that loss of Ron may sensitize cells to 1,25D_3_-mediated inhibition of growth and tumor progression. Furthermore, when R7 cells were transfected with a mutated β-catenin construct for enhanced activity, the effects of 1,25D_3_ on cell number were significantly reduced. Together, these data elucidate the potent ability of β-catenin signaling to evade the protective effects of vitamin D_3_ in Ron-overexpressing breast cancer cells, and suggest a role for dual-therapies targeting the β-catenin and/or Ron signaling pathways in combination with vitamin D_3_ or vitamin D_3_ analogs.

Active β-catenin protein levels were significantly higher in VDR deficient mammary glands, which correlated with elevated levels of cyclin D1 and c-Myc. Evidence of enhanced β-catenin transcriptional activity with loss of VDR was further substantiated by significantly elevated mRNA levels of β-catenin target genes cyclin D1, TCF-7 and MMP7. Similar to the *in vivo* studies with VDR deletion, loss of active β-catenin levels were observed in R7 cells treated with 1,25D_3_ that correlated with reduced expression of β-catenin target genes. Numerous studies have linked aberrant β-catenin signaling with epithelial to mesenchymal transition (EMT) and invasiveness in human cancer [38, 39]. It has recently been shown that VDR signaling inhibits epithelial to mesenchymal transition and the metastatic potential of human and mouse breast cancer cells induced by tumor associated macrophages *in vitro* and in an orthotopic model of breast cancer [33]. Moreover, VDR expression negatively correlates with human breast cancer metastasis. Although TCF-7 expression in breast cancer cells is largely unexplored, TCF-7 is activated in colorectal cancer, leading to metastatic behavior by overproduction of lymphoid tyrosine protein kinase p56 [40]. Increased TCF-7 gene expression in MMTV-Ron VDR−/− tumors and decreased expression in response to 1,25D_3_ in R7 cells may suggest a mechanism by which VDR mitigates metastatic burden in Ron-overexpressing breast cancers. However, future work is needed to verify the potential for reduced metastasis with VDR-mediated downregulation of TCF-7.

In normal and transformed epithelial cells, matrix metalloproteinases (MMPs) play important roles in cell migration, invasion, proliferation, apoptosis, angiogenesis, and host defenses through various mechanisms; mainly the regulation of ECM proteins in the microenvironment (reviewed in [41]). Interestingly, MMP7 can enhance the growth rate of normal mammary epithelial cells and promote tumor formation *in vivo* [42]. Enhanced expression of MMP7 in MMTV-Ron VDR−/− mammary tumors and, conversely, reduced expression in R7 cells treated with 1,25D_3_ suggests a mechanism by which ligand-dependent VDR signaling delays tumor formation and mitigates progression.

**Figure 7:**
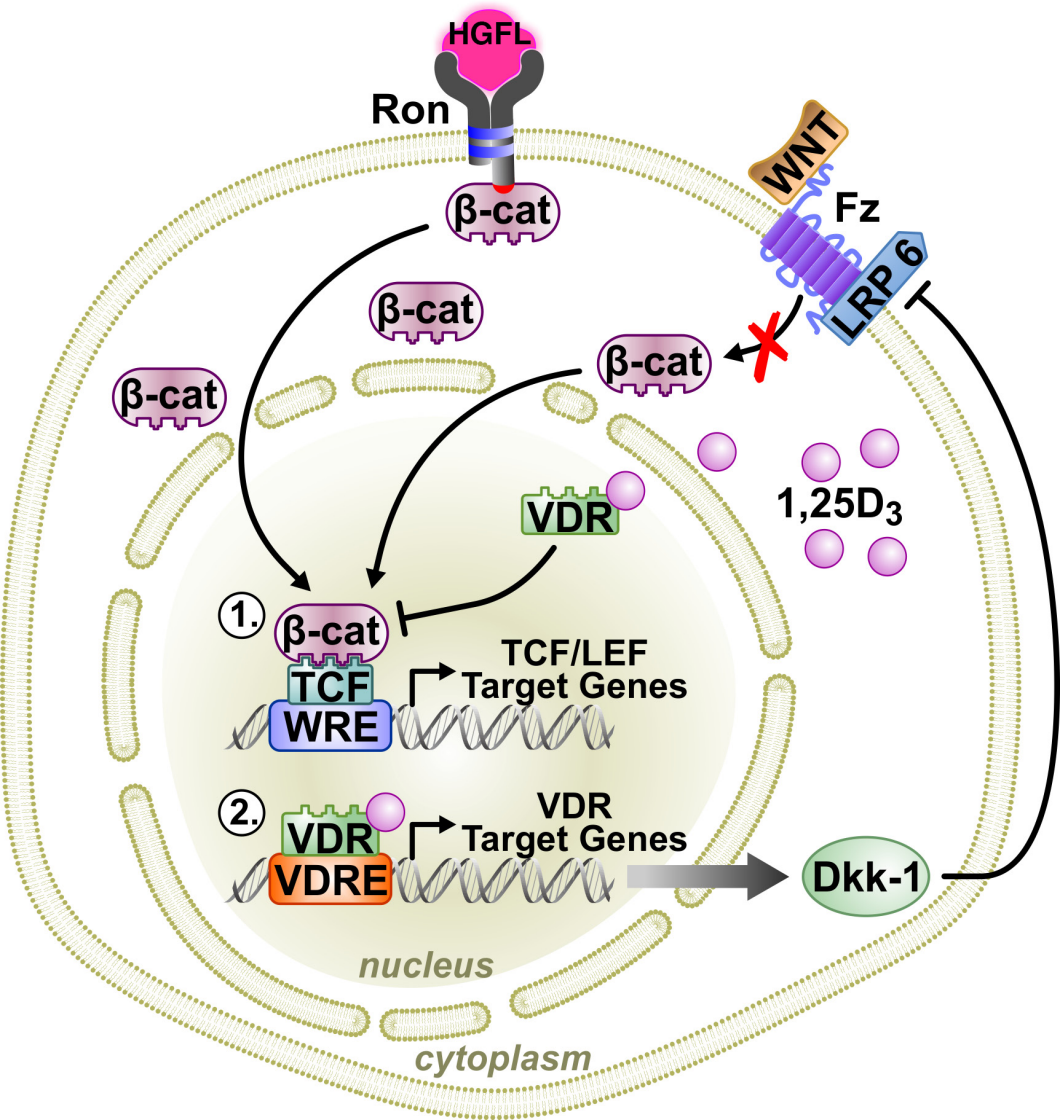
A working model of VDR signaling in Ron-mediated breast cancer Binding of the Ron ligand, HGFL, to Ron induces β-catenin activation promoting breast cancer growth and metastasis when aberrantly expressed. VDR signaling in breast cancer cells antagonizes β-catenin activity, delaying tumor initiation and progression through two mechanisms: 1) Vitamin D_3_-bound VDR can directly interact with β-catenin, preventing its association with TCF/LEF transcription factors thus inhibiting transcriptional activity; and, 2) ligand-dependent VDR signaling can regulate the transcription of various VDR target genes. One such VDR target includes DKK-1, which inhibits canonical Wnt signaling, thereby reducing cytosolic accumulation of β-catenin and nuclear localization for transcriptional activity.

Similar to colon cancer, 1,25D_3_ dose-dependently increased expression of the extracellular canonical Wnt inhibitor, DKK-1, in R7 cells while loss of VDR in MMTV-Ron mice reduced tumoral DKK-1 mRNA. Functionally, 1,25D_3_-stimulated DKK-1 expression was associated with growth inhibition. These data suggest a working model by which VDR inhibits Wnt/β-catenin signaling and breast cancer in MMTV-Ron mice through the transcriptional regulation of DKK-1 expression (Figure 7). DKK-1 suppresses β-catenin cytosolic accumulation, nuclear translocation and transcriptional activity by inhibiting the Wnt co-receptor low density lipoprotein receptor-related protein 6 (LRP6) from binding to Frizzled [43]. Interestingly, there is appreciable evidence of crosstalk between the Ron and canonical Wnt signaling pathways involving downstream activation of ERK [44], which can phosphorylate LRP6 for enhanced Wnt signaling [45] and inactivation of GSK-3β [46], both resulting in elevated levels of β-catenin. Additionally, previous studies have shown that oncogenic Ron can intensify β-catenin signaling through activation of dishevelled [12]. However, more work is needed to understand the interactions between the Ron and canonical Wnt signaling pathways in directing β-catenin activation.

Analogous to previous studies in colon cancer showing direct interaction of VDR and β-catenin [32, 47], we found that active β-catenin had significantly less interaction with the cyclin D1 promoter after 1,25D_3_ treatment in mammary tumor cells. Moreover, enrichment of the VDR/active β-catenin complex at the cyclin D1 promoter was also markedly reduced in the presence of 1,25D_3_. These data support a model suggesting that the ability of ligand-bound VDR to directly reduce β-catenin transcriptional activity is by sequestering β-catenin from its TCF/LEF target genes (Figure 7).

In summary, our data show that VDR plays a protective role in Ron-mediated breast tumorigenesis and progression by both direct and indirect mechanisms that antagonize β-catenin transcriptional activity. Many studies utilizing vitamin D_3_ [48, 49] or vitamin D_3_ analogs [50-54] have shown a protective role against breast cancer development, progression, and metastasis. The mechanisms include inhibition of angiogenesis [48], induction of cell cycle arrest at the G1/S checkpoint [49], promotion of apoptosis [50, 51], and reduced overall tumor growth [53]. Similarly, many therapeutic agents targeting Ron and/or other receptor tyrosine kinases have also demonstrated efficacy in preclinical and clinical trials [55]. These include monoclonal antibodies that neutralize HGFL-dependent Ron signaling [56-60], small molecule inhibitors targeting Ron [61], and the use of anti-Ron antibodies for the delivery of cytotoxic drugs in Ron-overexpressing cancers [62]. Results from Ron-targeted treatments have demonstrated reductions in cell migration *in vitro* [56, 58], decreased tumor growth *in vivo* [57, 59], and enhanced sensitivity to additional cytotoxic drugs [57, 60]. However, other studies have shown that singular therapies targeting Ron [56] or dietary supplementation with vitamin D_3_ (reviewed in [63-65]) is not sufficient to block tumor growth and progression. Aberrant downstream β-catenin activation with Ron overexpression is likely a strong contributor to tumorigenesis in the breast, although we recognize this pathway may not be the only tumor-driving mechanism. Also, due to the ability of 1,25D_3_/VDR signaling to regulate many cellular functions in the breast, we postulate that VDR-dependent suppression of Ron-induced mammary tumorigenesis is likely multifactorial and not necessarily limited to antagonizing the β-catenin pathway. However, our study herein strongly implicates the potential for combination therapies targeting Ron, or more specifically β-catenin signaling, and treatment with vitamin D_3_ or potent vitamin D_3_ analogs that may benefit patients with aggressive, Ron-mediated breast cancer.

## MATERIALS AND METHODS

### Mice

VDR knockout (VDR−/−) animals [66] were crossed to MMTV-Ron mice [13] to generate litters heterozygous for VDR. Littermates were then crossed to generate mice homozygous, heterozygous, or null for VDR all with Ron overexpression specific to the mammary epithelium. All animals were continuously maintained on a diet high in calcium (2%), phosphorous (1.25%), and lactose (20%) containing 2.2 IU Vitamin D_3_/g (Research Diets, New Brunswick, NJ). Two hours before sacrifice, mice were injected with 3 mg/mL BrdU. All mice were maintained under specific pathogen-free conditions and were treated and euthanized in accord with protocols approved by the University of Cincinnati Institutional Animal Care and Use Committee.

### Whole mount preparation

Inguinal mammary glands were isolated and processed as previously described [67]. Images were taken using Axiovision 4.5 Software (Jena, Germany). A minimum of 8 mice per genotype was used for verification of hyperplasia.

### Histological staining

Formalin-fixed, paraffin-embedded right inguinal mammary glands, lungs, liver, and tumors from 2, 4, 6, 8, 10 and 12-month mice were cut into 5 micrometer tissue sections and stained with hematoxylin and eosin Y for gross morphological assessment. Tissue sections from each mouse at each time point were assessed for hyperplasia in the mammary gland and metastasis in the lungs and liver. Metastases were verified histologically and by immunohistochemical staining for Ron expression. Mammary glands from eight month-old MMTV-Ron VDR+/+ and VDR−/− mice were assessed for β-catenin expression by immunohistochemistry. Briefly, antigen retrieval was performed in citrate buffer, tissues were quenched for endogenous peroxide, blocked in goat serum, and the primary antibody against β-catenin (Cell Signaling, Danvers, MA, 1:100) or Ron (Santa Cruz Biotechnology, Santa Cruz, CA, 1:100) was incubated at 4°C overnight. Detection of β-catenin or Ron staining was detected with 3,3′-diaminobenzidine (Sigma-Aldrich, St Louis, MO). Staining was analyzed at 40X magnification using ImageJ Software (National Institutes of Health, Bethesda, MD). A minimum of eight animals per genotype was evaluated with two sections per animal.

### Western blotting

Whole thoracic mammary glands from 6 month-old mice, R7 cells, T47D shNT cells or T47D shRon cells were homogenized in RIPA lysis buffer, sonicated, and measured for protein concentration using a BCA protein assay. Immunoblotting was carried out using antibodies targeting VDR-D6 (Santa Cruz), Ron (Santa Cruz), active β-catenin (ABC, clone 8E7, Millipore, Billerica, MA), total β-catenin (Cell Signaling), cyclin D1 (Cell Signaling), c-Myc (Cell Signaling), DKK-1 (Santa Cruz) and tubulin (Santa Cruz).

### ChIP/Re-ChIP

R7 cells treated with 100 nM 1,25D_3_ or EtOH (vehicle control) were fixed in 37% formalin, digested and sheared to a length between 200 and 2000 bp. Lysates were precleared with mouse IgG and immunoprecipitated with 5 μg of an anti-active β-catenin antibody (ABC) or anti-active β-catenin followed by secondary immunoprecipitation with 5 μg of an anti-VDR (D6) antibody. Chromatin and protein crosslinks were removed with 5M NaCl and DNA was purified using the QIAquick PCR Purification Kit (Qiagen, Venlo, Limburg) according to the manufacturer's instructions. Primer sets designed to encompass the β-catenin/LEF-1 recognition sequence in the mouse cyclin D1 promoter were utilized for qRT-PCR for examination of interactions with active β-catenin. The following primer sequences were used: (5′–TTCTCTGCCCGGCTTTGAT–3′) and (5′– AACTTCAACAAAACTCCCCTGTAGTC–3′); and to an ‘off-target’ site without a β-catenin binding element approximately 4000 bp upstream, within the cyclin D1 promoter: (5′-AGAGACAGGCATCCCATTGGT–3′) and (5′-CAGGTCAGAGTGCTTGCTGATT–3′).

### Quantitative real time PCR (qRT-PCR)

Total RNA was isolated from whole mammary glands, R7 cells, or human T47D cells using Tri-Reagent RT (Molecular Research Center, Inc., Cincinnati, OH) and subsequently reverse transcribed into cDNA (1.5 μg) using the High Capacity cDNA Reverse Transcription Kit (Applied Biosystems, Foster City, CA). Between four and eight experimental cDNA samples were analyzed for VDR, Ron, β-catenin, cyclin D1, c-Myc, MMP7, TCF-7, VEGF, and DKK-1 mRNA expression in duplicate by real time PCR analysis using FastStart SYBR Green Master (Roche Diagnostics, Indianapolis, IN). The following forward and reverse mouse primer sets were used: VDR (5′–GAAGCGCAAGGCCCTGTT–3′) and (5′–CGCTGCACCTCCTCATCTGT–3′); Ron (5′–TCCCATTGCAGGTCTGTGTAGA–3′) and (5′–CGGAAGCTGTATCGTTGATGTC–3′); β-catenin (5′–TCCCTGAGACGCTAGATGAGG–3′) and (5′– CGTTTAGCAGTTTTGTCAGCTC–3′); cyclin D1 (5′–GCGTACCCTGACACCAATCTC–3′) and (5′–CTCCTCTTCGCACTTCTGCTC–3′); c-Myc (5′–TAACTCGAGGAGGAGCTGGA–3′) and (5′–GCCAAGGTGTGAGGTTAGG–3′); TCF-7 (5′–AGCATCCGCAGCCTCAAC–3′) and (5′–AGCGGCCTGTGAACTCCTT–3′); VEGF (5′–GCAGAAGTCCCATGAAGTGA–3′) and (5′–TCCAGGGCTTCATCGTTA–3′); MMP7 (5′–CTGCCACTGTCCCAGGAAG–3′) and (5′–GGGAGAGTTTTCCAGTCATGG–3′); DKK-1 (5′– CCTTCGGAGATGATGGTTGT–3′) and (5′–GTTCTGATCGCGTTGGAAT–3′). Gene expression was normalized to 18S rRNA and reported as relative expression compared to the wild type control.

### Cell culture

R7 murine breast cancer cells were derived from a mouse mammary tumor from MMTV-Ron mice as previously described [13]. Human T47D cells were purchased from American Type Culture Collection (Manassas, VA) were previously infected with a non-target (T47D shNT) control or Ron shRNA lentivirus (cat#RHS3979-9571732) purchased from Open Biosystems (Huntsville, AL). Polyclonal stable cell lines were generated as previously described [14].

### Dual luciferase assay

R7 cells were dual-transfected with the TCF reporter plasmid TOP-FLASH (2 μg; Upstate Biotechnology, Lake Placid, NY, USA) expressing ﬁreﬂy luciferase and 2 sets of 3 copies of the TCF binding site, and pRLTX expressing *Renilla* luciferase (0.04 μg; Clontech, Mountain View, CA) with Lipofectamine 2000 (Invitrogen, Carlsbad, CA) for 48 h. The cells were then treated with 1,25D_3_ (100 nM) for 24 hours followed by determination of luciferase activity according to the manufacturer's instructions (Promega, Madison, WI). Values were normalized to *Renilla* luciferase expression.

### Cell viability/MTT/crystal violet

R7 and T47D cells were seeded in a 12-well or 24-well plate, respectively, at a density of 5,000 (R7) or 25,000 (T47D and T47D shRon) cells per well and allowed to adhere overnight. Fresh media was placed onto the cells with or without the indicated concentration of 1,25D_3_. After 48 hours, the media was aspirated from T47D cells and 1 mg/ml thiazolyl blue tetrazolium bromide (Sigma-Aldrich) was added for 60 minutes prior to incubation with DMSO. Absorbance was measured at 570 nm. After 72 hours, the media was aspirated from R7 cells which were then washed with 1X PBS, fixed in 4% paraformaldehyde for 10 minutes, and stained with crystal violet for 60 minutes. Images were taken at 20X using Axiovision 4.5 Software (Jena, Germany) and viable cells were counted manually using ImageJ Software (National Institutes of Health).

### Transfection with β-catenin constructs and siRNA

R7 cells were transfected with a plasmid construct (5 μg/1:10^6 cells) containing a stabilized form of β-catenin (ΔN) [34] using the Amaxa Nucleofector 2b Device (Lonza, Alpharette, GA) according to the manufacturer's instructions. Transfection efficiency was roughly 35% in R7 cells (data not shown). The cells were treated with 1,25D_3_ (100 nM) for 72 hours followed by determination of cell number using a crystal violet assay. DKK-1 siRNA (25 nM; Santa Cruz) was transfected into R7 cells using the Amaxa Nucleofector 2b Device according to the manufacturer's instructions. Targeted DKK-1 knockdown was confirmed via Western blot 48 hours post transfection. Cells were utilized for cell growth/viability assays with or without 1,25D_3_ as described above.

### Scratch/cell migration assay

R7, T47D, and T47D shRon cells were seeded in a 6-well plate labeled with a vertical line across the diameter of each well and seeded at a density of 50,000 (R7) or 100,000 (T47D and T47D shRon) cells/well. Cells were grown to 80% confluence then three horizontal scratches perpendicular to the labeled diameter line were inscribed across each well using a sterile p-200 pipette tip. The media was changed and 1,25D_3_ or EtOH (vehicle control) was added to each well at the designated concentration. Images were taken at 5X magnification at 0, 4, 8, and 24 hours after treatment. The length of each scratch was calculated from three measurements per image and percent wound closure was measured using ImageJ Software (National Institutes of Health). Cell number was also measured via MTT (as described above) at the designated time points to account for differences in growth rate.

### Cell invasion/matrigel assay

R7 cells were seeded at a density of 50,000 cells/well in 6.5 mm transwell inserts (8.0 μm pore size) in a 24-well plate above a 70 μL-thick growth factor reduced matrigel matrix (BD Biosciences, San Jose, CA). Cells were plated in serum-free media in the presence of 100 nM 1,25D_3_ or EtOH (vehicle control). Below the insert, culture media containing 10% FBS was added as a chemoattractant. Cell invasion was quantified after 48 hours by measuring the viability (MTT) of cells that had traversed the matrigel matrix.

### Statistical analyses

Statistical analysis was conducted using GraphPad Prism software (La Jolla, CA). For observance of mammary gland hyperplasia, Chi-square analysis was utilized for identifying differences between MMTV-Ron VDR+/+ and MMTV-Ron VDR−/− mice. Student's t tests or analysis of variance (ANOVA) tests were applied to determine significant differences. Data are expressed as the mean ± standard error (SE). A P-value <0.05 was regarded as significant.
